# Supplementary material for: Hepatitis B virus infection status and associated factors among health care workers in selected hospitals in Kisumu County, Kenya: A cross-sectional study
Source: PLOS Glob Public Health. 2023 Sep 20;3(9):e0001535. doi: 10.1371/journal.pgph.0001535 (PMC10511138; doi:10.1371/journal.pgph.0001535)
Supplement: Attachment — Submitted filename: Response to Reviewers.pdf [file pgph.0001535.s001.pdf]

Dear Academic Editor
PLOS Global Public Health

I wish to thank you for taking time to review the manuscript entitled ***Hepatitis B virus infection status and associated factors among health care workers in selected hospitals in Kisumu County, Kenya: a cross-sectional study.***
I have responded to the reviewers' comments as highlighted in the below table.

S/No	Suggested Observation(s)	Correction/Response
1	**Introduction**<br>I suggest including the vaccination strategy for HBV in Kenya (general population and healthcare workers).	This was added in the revised manuscript introduction section.
2	Methods<br>Can you Detail the method used to choose the 192 HCWs? How many illegible health professionals were in each Health Unit and how were the 192 chosen??<br>The study was conducted from May 2020 to April 2021. What was the recruitment period?<br>Where were the sample tests performed? How was the quality of results ensured when testing for HBV serological markers?	The sampling method has been added in the method section, the total number of HCWs has been added.<br>The recruitment period has been added, the location of test and measures to ascertain quality of results have been added.
3	Results<br>Suggest organizing table 2 into HCW infected (HBsAg positive), susceptible (negative for all markers), with immunity due to vaccination (Anti-HBs only), and immunity due to past infection (Anti-HBc and Anti-HBs). This change will affect the presentation of the results in the abstract, "prevalence of HBV Biomarkers" and discussion. How many HBsAg positive participants had two and three doses of vaccine? Separating into two categories (with at least one dose of vaccination/unvaccinated) can help reduce the amplitude of the confidence interval.	Table 2 has been separated to reflect to suggested changes. The results and discussion have been updated to reflect the changes.<br>Table 3 had been updated to separate dosage of Hepatitis B vaccination.<br>All the tables and figures has been formatted to show clarity.
4	Discussion<br>In the discussion of the anti-HBs results, I additionally suggest a separate discussion of immunity by vaccination.<br>It is interesting that a recently published study on HBV prevalence in HCWs in Mozambique also found high numbers of vaccinated laboratory technologists with the same justification. I suggest adding it to the discussion (line 261).<br>Correct the sentence in lines 273-274.<br>Correct the sentence in line 297 "facility related"	The discussion on vaccination has been added, the study in Mozambique has been added and cited<br>The sentences in role 273-274 and 297 has been corrected
5	Conclusion<br>I suggest that the conclusion be summarized, with a focus on the significance and implication of the results found.	The conclusion has been rewarded

Looking forward for your positive response.

Kind regards.

Frankline Otieno Mboya.
