# Supplementary material for: Hepatitis B virus infection status and associated factors among health care workers in selected hospitals in Kisumu County, Kenya: A cross-sectional study
Source: PLOS Glob Public Health. 2023 Sep 20;3(9):e0001535. doi: 10.1371/journal.pgph.0001535 (PMC10511138; doi:10.1371/journal.pgph.0001535)
Supplement: Attachment — Submitted filename: Review report.docx [file pgph.0001535.s002.docx]

**Review report: Hepatitis B virus Manuscript**

Thank you for sharing the manuscript titled "Hepatitis B virus infection status and associated factors among health care workers in selected hospitals in Kisumu County, Kenya: a cross-sectional study."

Please find my comments below;

**Abstract:**

**Line 31:** 192 HCW from nine purposively selected high-volume public hospitals is a very small sample size for generalization

**Line 31:** Need to specify the H.C.W.s (Physician, Nurses, Cleaning staff etc).

**Line 36:** Marital status is not essential s\o remove

**Line 41: Better to s**tart with the prevalence rate **then** vaccination status

Also, it should include the needle stacks injuries.

Line 50: Conclusion should be based on findings with practical recommendation

**Background:**

The background should highlight the global burden (both Developed and Developing countries), regional burden, and then local burden,

Line 78: Better to use references/ evidence from similar settings, you can use the following publication: Harun, M.G.D., Sumon, S.A., Mohona, T.M., Rahman, A., Abdullah, S.A.H.M., Islam, M.S. and Anwar, M.M.U., 2022. Hepatitis B Vaccination Coverage among Bangladeshi Healthcare Workers: Findings from Tertiary Care Hospitals. Vaccines, 11(1), p.41.”

**Methods**

Line 92: Need to mention the healthcare settings, patient flow, and patient turn over

Line: 96: The study population and characteristic of the participants and description is not consistent

Line 97: Need to specify the H.C.W.s, total number of H.C.W.s, and what % has been enrolled in this study.

Line 107: Data and sample collection should be the sub-title and description accordingly

I think this is not the updated version of the manuscript, as there is a lot of error and crack changes (deleted) in tables and texts. Recommend sharing the updated one with no track change error.
