# Supplementary material for: Hepatitis B virus infection status and associated factors among health care workers in selected hospitals in Kisumu County, Kenya: A cross-sectional study
Source: PLOS Glob Public Health. 2023 Sep 20;3(9):e0001535. doi: 10.1371/journal.pgph.0001535 (PMC10511138; doi:10.1371/journal.pgph.0001535)
Supplement: Attachment — Submitted filename: Response to Reviewers.docx [file pgph.0001535.s003.docx]

Dear Academic Editor
PLOS Global Public Health

I wish to thank you for taking time to review the manuscript entitled ***Hepatitis B virus infection status and associated factors among health care workers in selected hospitals in Kisumu County, Kenya: a cross-sectional study.***
I have responded to the reviewers’ comments as highlighted in the below table.

| **S/No** | **Suggested Observation(s)** | **Correction/Response** |
| --- | --- | --- |
| 1 | **Abstract:**  **Line 31:** 192 HCW from nine purposively selected high-volume public hospitals is a very small sample size for generalization.  **Line 31:** Need to specify the H.C.W.s (Physician, Nurses, Cleaning staff etc).  **Line 36:** Marital status is not essential s\o remove.  **Line 41: Better to s**tart with the prevalence rate **then** vaccination status.  Also, it should include the needle stacks injuries.  Line 50: Conclusion should be based on findings with practical recommendation. | The Abstract sections have been revised as per reviewer suggestions.  Sections recommended for removal i.e the marital status have been removed.  **Limitation section has been edited to highlight the sample size and generalization.** |
| 2 | The background should highlight the global burden (both Developed and Developing countries), regional burden, and then local burden,  Line 78: Better to use references/ evidence from similar settings, you can use the following publication: Harun, M.G.D., Sumon, S.A., Mohona, T.M., Rahman, A., Abdullah, S.A.H.M., Islam, M.S. and Anwar, M.M.U., 2022. Hepatitis B Vaccination Coverage among Bangladeshi Healthcare Workers: Findings from Tertiary Care Hospitals. Vaccines, 11(1), p.41. | **Yes, section revised and suggested citations inserted** |
| 3 | Methods Line 92: Need to mention the healthcare settings, patient flow, and patient turn over.  Line: 96: The study population and characteristic of the participants and description is not consistent  Line 97: Need to specify the H.C.W.s, total number of H.C.W.s, and what % has been enrolled in this study.  Line 107: Data and sample collection should be the sub-title and description accordingly:  I think this is not the updated version of the manuscript, as there is a lot of error and crack changes (deleted) in tables and texts. Recommend sharing the updated one with no track change error. | **This section has been revised for consistency and to give more clarity on the settings, patient flow, turnover and suggested subtitles have been added. Both the clean and revised manuscript have been uploaded** |
| 4 | Please upload all main figures as separate Figure files in .tif or .eps format only and remove them from the manuscript file. | **Figure 1 has been converted to TIF and submitted separately** |

Looking forward for your positive response.

Kind regards.

Frankline Otieno Mboya.
